# Nodal Cellular Blue Nevus in Sentinel Lymph Node Biopsy: A Case Report With Emphasis on Avoiding Misdiagnosis of This Important Mimicker of Metastatic Melanoma

**DOI:** 10.1111/cup.70006

**Published:** 2025-11-03

**Authors:** Kathie Velez, Kimberly Breglio, Robin T. Petroze, Simon J. Warren, Paul W. Harms, Kelly L. Harms, Jaclyn M. Plotzke

**Affiliations:** ^1^ Department of Dermatology Michigan Medicine and University of Michigan Medical School Ann Arbor Michigan USA; ^2^ Department of Pediatric Surgery Michigan Medicine and University of Michigan Medical School Ann Arbor Michigan USA; ^3^ Department of Pathology Michigan Medicine and University of Michigan Medical School Ann Arbor Michigan USA

**Keywords:** melanoma, melanoma arising in blue nevus, melanoma ex‐blue nevus, nodal blue nevus, nodal cellular blue nevus, nodal nevus

## Abstract

Melanoma arising in blue nevus (MBN) is a rare, aggressive malignancy that can develop from a preexisting blue nevus or resemble a cellular blue nevus without a clear precursor. We present a diagnostically challenging case of MBN on the foot of a 13‐year‐old female, with two sentinel lymph nodes (SLNs) showing heavily pigmented cells within the capsule and trabeculae. In contrast to the primary tumor, the cells within the SLNs contained bland spindled to epithelioid melanocytes with low proliferation and no significant cytologic atypia. Chromosomal microarray analysis was utilized to compare genetic profiles in both the primary melanoma as well as the lymph node. The melanoma revealed five copy number aberrations. Conversely, the SLN exhibited a normal profile. The findings were compatible with multiple nodal cellular blue nevi. The distinction between metastatic melanoma and nodal nevus is an important one with significant clinical implications. This case underscores the diagnostic challenge nodal cellular blue nevi can pose and the importance of correlating morphology, immunophenotype, and in difficult cases, molecular findings, to avoid misdiagnosis as metastatic melanoma. In particular, array‐based comparative genomic hybridization (aCGH) was helpful in this case.

## 1 Introduction

Melanoma arising in blue nevus (MBN), also known as melanoma ex‐blue nevus, is a malignant melanocytic proliferation arising from a preexisting blue nevus or at the site of a previously excised blue nevus/cellular blue nevus. The term also encompasses melanoma which morphologically resembles cellular blue nevus, but without an identifiable benign precursor [1]. Blue nevi and related lesions arise from mutations in the Gαq pathway, including *GNAQ, GNA11, PLCB4*, or *CYSLTR2* mutations [2, 3, 4, 5]. A recent retrospective cohort analysis of the National Cancer Database of 118 patients diagnosed with MBN showed the average age of diagnosis to be 56.7 ± 17.2 years with female predominance; over half presented on the scalp, neck, or trunk [6]. The distinction between entities in the spectrum of blue nevus to MBN is notoriously challenging. While MBN is much rarer than its benign counterpart, it is essential for accurate diagnosis as there is often an aggressive clinical course with frequent metastasis to lymph nodes and visceral organs [1].

Evaluation of sentinel lymph nodes (SLN) is an important component of melanoma staging. In approximately 20% of SLN specimens, benign nodal nevi are identified [7, 8]. A recent retrospective observational study found the sentinel nodes of three out of four patients with malignant blue nevus displayed nodal nevi [9]. The diagnosis of metastatic melanoma and nodal nevus is often straightforward, especially with the help of immunohistochemistry. However, there are cases in which the nevus cells display unusual features, creating diagnostic uncertainty with significant clinical implications for the patient. Nodal nevi resembling blue nevi have rarely been reported [10, 11, 12, 13, 14, 15, 16].

We present a histologically challenging case of melanoma arising in blue nevus on the foot of a 13‐year‐old female with two (2) SLNs showing robust nodal cellular blue nevi. We aim to emphasize important morphologic and molecular features to avoid misdiagnosis of nodal cellular blue nevus as metastatic melanoma.

## 2 Case Report

A 13‐year‐old Hispanic female with no significant past medical history presented with an enlarging 1.5 cm black‐to‐blue patch with a centrally elevated verrucous papule on the left dorsal foot that has been present since birth.

**FIGURE 1:**
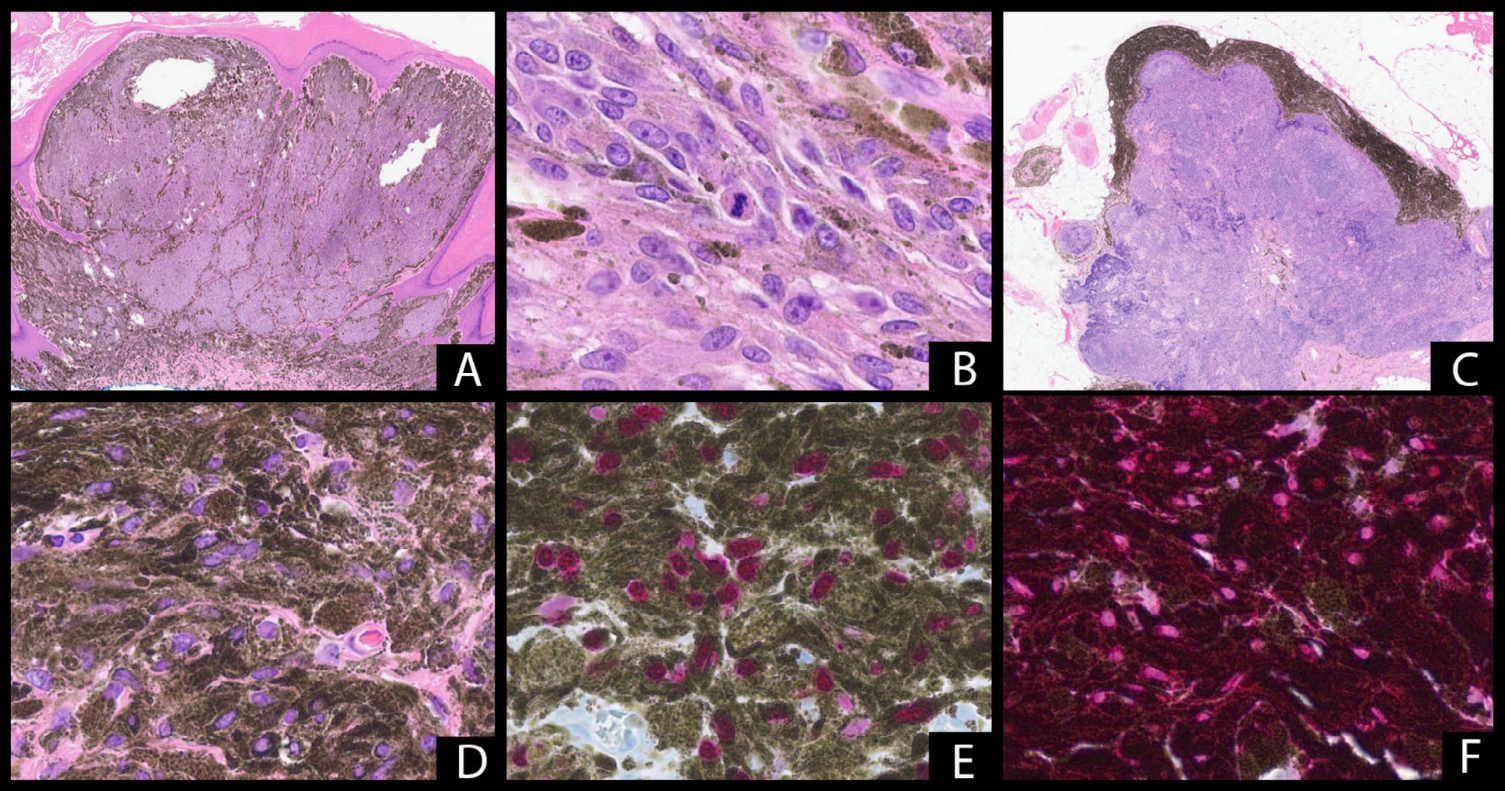
Primary lesion demonstrating expansile and exophytic growth (A, H&E, ×14). High‐power view of primary lesion exhibiting cytologic atypia, cells with melanized cytoplasm, mitotic activity. No necrosis was identified (B, H&E, ×400). Sections of lymph node showing pigmented cells in a predominantly capsular and trabecular pattern (C, H&E, ×15). High‐power view of lymph node capsule demonstrating ovoid cells with pigmented cytoplasm without significant cytologic atypia or mitotic activity (D, H&E, ×400). Melanocytes within the lymph node capsule are highlighted by SOX10 immunohistochemistry (E, SOX10, ×400). Ki67 (brown)/MART1 (red) dual stain on lymph node. Cells are highlighted by MART1 with low overall proportion of nuclei staining with Ki57. Background melanin pigment (F, Ki67/MART1, ×400).

Sections demonstrated a biphenotypic dermal melanocytic proliferation. The first population showed expansile exophytic nodules of large cells with pleomorphic nuclei, prominent nucleoli, hyperchromasia, melanized cytoplasm, and readily identifiable mitotic figures (Figure 1A,B). No necrosis was identified. The second morphologic population, present at the base and periphery, consisted of mildly enlarged pigmented dendritic melanocytes dispersed at a moderate density within sclerotic stroma. By immunohistochemistry, the cells showed diffuse HMB45 labeling and an elevated Ki67 proliferation index (via Ki67/MART1 dual stain) within the expansile population. BAP1 was retained, and PRAME was negative for overexpression.

Array‐based comparative genomic hybridization (aCGH) was conducted using the OncoScan SNP array (as previously described [17, 18]) and analyzed using Chromosome Analysis Suite v4.4.0.63 (Thermo Fisher Scientific). Results showed 5 abnormalities including gains in 1q, 7p, 8q (encompassing MYC), and 18p, and a loss in 8p. Next generation sequencing (Oncomine Focus Assay 50 gene panel, ThermoFisher) demonstrated the *GNAQ* p. Q209L hotspot mutation. The morphologically distinct cell population with cytologic atypia, elevated proliferation index, dermal mitotic figures, and molecular findings was most consistent with melanoma ex‐blue nevus, invasive to a Breslow depth of 2.9 mm.

**FIGURE 2:**
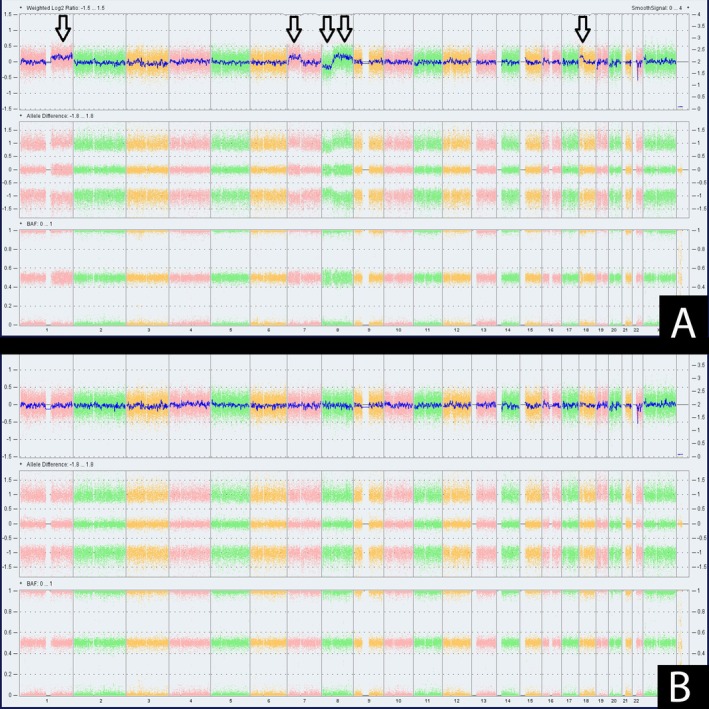
Array CGH corresponding to primary lesion, showing five chromosomal aberrations delineated with arrows (A). Array CGH of lymph node specimen showing a normal profile in contrast to the abnormal profile detailed above (B). BAF, biallelic frequency.

The patient subsequently underwent wide local excision and additional staging with SLN biopsy. SLNs were grossly abnormal in color and size. Both SLNs showed heavily pigmented spindled dendritic to epithelioid cells present predominantly within the lymph node capsule and trabeculae (Figure 1C). The cells of interest were highlighted by MelanA, S100, and SOX 10 with an overall low proliferation index by Ki67/MART1 dual stain (Figure 1D–F). Given the diagnostic challenge of distinguishing robust cellular capsular blue nevus from metastatic melanoma and the significant associated clinical implications, chromosomal microarray analysis was also performed on the lymph node specimen. aCGH demonstrated a normal profile in contrast to the five abnormalities shown on the skin biopsy (Figure 2A,B). Morphologic, immunophenotypic, and molecular findings in the lymph nodes were compatible with nodal cellular blue nevus. No additional foci diagnostic of metastatic melanoma were identified.

## 3 Discussion

Blue nevi are dermal melanocytic proliferations composed of spindled and dendritic cells. Cellular blue nevi are typically > 1 cm in size, nodular, and often located on the scalp or sacrococcygeal area, while common blue nevi are smaller and frequently found on the dorsal distal extremities. Most harbor *GNAQ* or *GNA11* mutations, linking them genetically to uveal melanoma [19]. Melanoma ex‐blue nevus is a malignant transformation, often with an associated benign precursor, frequently characterized by nuclear pleomorphism, coarse chromatin, prominent nucleoli, atypical mitoses, tumor necrosis, and invasive growth [20]. This spectrum of melanocytic lesions is notoriously diagnostically challenging for a variety of reasons. For instance, the subjectivity and lack of consensus on what constitutes malignancy and small biopsies with incomplete sampling of lesions are obstacles to definitive diagnosis. Interestingly, in a study of 24 patients with melanoma ex‐blue nevus, 15 had SLN biopsies, yet none were found to have nodal blue nevi [20].

The overall frequency of nodal nevi (including blue nevi) detected in SLN biopsies has increased—ranging from 1% to 22%—since the adoption of SLN mapping in breast cancer and melanoma [7, 21]. The International Collaboration on Cancer Reporting, the College of American Pathologists, and the Royal College of Pathologists of Australasia emphasize careful assessment of location, cytologic features, and immunohistochemical staining to assist in the distinction from metastatic melanoma [22]. Nodal nevi are composed of small, cytologically bland melanocytes with low mitotic activity, typically located in the capsule or trabeculae, and retain markers such as p16. Whereas metastatic melanoma often will be located within the subcapsular sinus and parenchyma, shows cytologic atypia, a higher mitotic rate, loss of p16, and an elevated Ki67 proliferation index (usually > 2%) [21, 22, 23]. Occasionally, nodal nevi can resemble blue nevi [10, 11, 12, 13, 14, 15, 16, 24]. Nodal blue nevi are established as benign lesions with indolent clinical behavior and no evidence of recurrence or metastasis on long‐term follow‐up, requiring no further treatment beyond excision, and long‐term surveillance is not indicated in the absence of atypical features or malignant transformation [25].

Often, the separation of nodal nevus from metastatic melanoma is made possible by the evaluation of morphologic and immunohistochemical features. However, in certain cases such as the current case, molecular techniques such as aCGH have become increasingly valuable adjuncts to traditional histopathologic and immunohistochemical methods. aCGH has a high sensitivity and specificity in separating benign melanocytic lesions from melanoma by detecting copy number gains and losses [18, 26, 27, 28]. Blue nevus‐like lesions have been investigated by this methodology and unequivocally benign cellular blue nevi reliably demonstrate no copy number alterations, whereas malignant lesions (often displaying 3 or more mitoses/10 hpf) have shown three or more chromosomal aberrations [29]. An additional study notably expanded the knowledge of copy number alterations in the atypical cellular blue nevus category [30]. Twenty‐five percent of atypical cellular blue nevi displayed copy number aberrations, yet none of the atypical cellular blue nevi showed more than one aberration. In contrast, complex alterations of four or more regions were seen exclusively in melanoma arising in blue nevus [30]. As was identified in the current case, gains of 8q have been frequently identified in MBN, among other recurrent and nonrecurrent alterations [3, 30]. Although aCGH can be informative in the distinction of metastatic melanoma from unrelated second melanocytic tumors [31], the use of aCGH in SLNs to assist in the distinction between nodal nevus and metastatic melanoma has not yet been well described in the literature.

In summary, the current case highlights the diagnosis of melanoma arising in blue nevus with subsequent SLN biopsy demonstrating nodal cellular blue nevus. Several morphologic characteristics were informative in the lymph node lesion, such as location within the capsule and trabeculae, lack of significant cytologic atypia, and lack of significant mitotic activity, to support the impression of nodal cellular blue nevus. However, given the robust appearance and involvement of multiple lymph nodes, aCGH was performed in comparison to that of the primary melanoma to assist in classification as nodal cellular blue nevus.

Accurate discrimination of benign nodal nevi from metastatic melanoma is an essential part of the assessment of SLN biopsies. Misdiagnosis of nevus as melanoma can lead to unnecessary upstaging, overtreatment, and psychological distress for the patient. Conversely, inaccurate diagnosis of melanoma as nevus can deprive a patient of essential early intervention. Therefore, we emphasize the importance of integrating histomorphologic findings (including comparison of the nodal cells to the primary lesion if available), architectural arrangement, immunohistochemistry, and occasionally molecular (optimally aCGH) modalities to avoid misdiagnosis of an important mimicker of metastatic melanoma.

## Ethics Statement

The authors have nothing to report.

## Conflicts of Interest

The authors declare no conflicts of interest.

## Data Availability

The data that support the findings of this study are available from the corresponding author upon reasonable request.
